# Internet Addiction, Sleep Habits and Family Communication: The Perspectives of a Sample of Adolescents

**DOI:** 10.3390/healthcare11243194

**Published:** 2023-12-18

**Authors:** Francisca Monteiro, Margarida Simões, Inês Carvalho Relva

**Affiliations:** 1 Department of Education and Psychology, University of Trás-os-Montes and Alto Douro, 5000-801 Vila Real, Portugal; francisca9monteiro@gmail.com (F.M.); margaridas@utad.pt (M.S.); 2 Centre for Research and Intervention in Education (CIIE), University of Porto, 4200-135 Porto, Portugal; 3 Research Center in Sports Sciences, Health Sciences and Human Development (CIDESD), University of Trás-os-Montes and Alto Douro, 5001-801 Vila Real, Portugal

**Keywords:** internet addiction, sleep habits, family communication, adolescence

## Abstract

Background: With the increase in communication technologies, the internet has become an indispensable tool in the life of the individual. Several studies report on the advantages of this resource; however, there is still a group of individuals who use the internet excessively. The aim of this study was to explore the relationships between internet addiction, daytime sleepiness, and family communication in adolescents. Methods: A total of 340 adolescents aged between 12 and 17 years participated in this study. All completed the sociodemographic questionnaire, the internet addiction test, the pediatric daytime sleepiness scale, and the family communication scale. Results: The results indicate that 64.1% of the adolescents had mild to moderate addiction to the internet. The main results suggest that internet addiction in adolescents is negatively associated with family communication and positively associated with excessive daytime sleepiness. It was also observed that gender had a significant effect on daytime sleepiness, with female participants having more excessive daytime sleepiness. Regarding age, the results indicate higher values of internet addiction among younger adolescents. Conclusions: In view of the above, it is considered important to develop preventive actions with a view to healthy family communication, with the adoption of sleep hygiene habits and the promotion of healthy use of the internet.

## 1. Introduction

Internet use has increased in recent years [1], affecting the entire population, especially younger people [2]. Nowadays, society has come to be known as the “digital society”, where new technologies are present in people’s daily lives [3]. Worldwide, according to Internet World Stats [4], more than 67.9% of the population has access to the internet, with Europe in second place with an internet penetration rate of 89.2%. In Portugal, in 2022, 88.2% of households had access to the internet [5] and, according to data from Pordata [6], 84.5% of Portuguese individuals aged between 16 and 24 use the internet, with males (85.5%) using it slightly more than females (83.6%).

With its increased use, a new concept associated with uncontrolled internet use was born, namely addiction, which has become a public health issue since 1990 [7]. The concept of “internet addiction” was first used in 1995 by Graham, and he describes it as a behavioral addiction that leads to an inability to control internet use, which leads to pathological suffering, denial of the problematic behavior, dysfunction in daily activities, isolation, mood swings, tolerance, and relapses [8]. In 1998, Young first used the term “internet addiction” and characterized it as a set of behaviors and impulse control disorders that are not related to chemical dependency [9]. Until then, no consensus had been determined regarding the terminology of the phenomenon of internet addiction, given that the scientific community uses different terms to characterize it [10]. Currently, the scientific community uses the term internet addiction; however, there are also researchers who use “Problematic Internet Use” (PIU) or “Internet Addiction Disorder” [1]. The term “Internet Addiction Disorder” is used to emphasize the severity of this condition and, therefore, the authors use it as a formal disorder classification, while “internet addiction” is used more broadly [11].

Although the term internet addiction is widely used in the scientific literature, it has not yet been recognized in the DSM-5 [12] as a dependence disorder and is placed in a separate chapter titled “Conditions for further study” [1]. This diagnostic manual only includes a behavioral addiction known as internet gaming disorder [13]. Likewise, in ICD-11, the pathology is called “Gaming disorder” and is divided into two groups, that is, predominantly online or offline [14].

Unlike other addictions, the internet brings benefits and advances to our society, making it a normalized addiction for individuals [15], as it is used at work, in the teaching–learning process, in social communication, and in the search for information [16]. However, some of the consequences associated with permanent internet connection are mostly related to the development of the risk of behavioral addiction, such as mood disorders, irritability, depression, sleep disturbances, loss of concentration, emotional fragility, social isolation, and others [17].

During adolescence, young people go through various transformations in their physical, cognitive, psychosocial, and sexual development, as well as identity crises [18]. Internet addiction in adolescents has consequences for their daily activities, threatens and damages family relationships [19], and makes them more introverted and more likely to have behavioral problems [20]. For this reason, internet addiction is more than abusive consumption, as it has specific clinical criteria that compromise the adolescent’s life, requiring urgent professional intervention [21].

Sleep has an important physical and psychological role in human quality of life, and healthy sleep habits are fundamental for adolescents, as they are in the growth and development phase [22]. When adolescents have a short sleep duration, changes occur in their daily activities and behavior, contributing to a decline in health, fitness, nutrition, and relaxation [23]. In fact, adolescents are naturally more vulnerable to sleep disorders, especially insomnia, since puberty is a time when melatonin is released, which leads to changes in the circadian rhythms of young people [24].

Sleep deprivation causes excessive fatigue, impairs academic performance, and increases the risk of poor diet and lack of physical exercise [15]. In addition, it can seriously interfere with adolescent development processes, reducing energy, concentration, and memory consolidation [25]. Sleep is fundamental to adolescent development and is essential for emotional, physical, and cognitive well-being, allowing the human body to regain the strength it needs to function optimally [26]. Thus, sleep deprivation is associated with various health problems, such as diseases of the renal and cardiovascular systems, and can also contribute to the development of metabolic abnormalities such as obesity, diabetes, and mental disorders [27]. Sleep deprivation affects human abilities with impairments in cognitive functions, emotional processes, muscular activity, and actions that require the activity of brain regions (prefrontal cortex, thalamus, and hippocampus), affecting learning processes and consolidation of memory [28].

The use of technology affects the onset and duration of sleep, which leads adolescents to develop daytime sleepiness, tiredness, and insomnia that can result in decreased school performance, poor concentration, and often school failure [29]. Daytime sleepiness is increasingly common in adolescents [30] and tends to increase over the years [31]. During the day, it is normal for there to be events that cause sleepiness in individuals; however, if this behavior becomes recurrent, it can lead to unsatisfactory levels of sleep and related disorders [32]. Daytime sleepiness is defined as the accumulated need for sleep and decreased alertness, one of the main causes of which is reduced sleep duration during the night [33].

The family is the place where adolescents build their personality through everyday family dynamics, like family communication [34]. Family communication patterns are established in early childhood and shape the cognitive schemas that will later influence the way individuals behave and interact [35]. During adolescence, parents’ interaction with their children takes on a different dimension, as young people seek autonomy and identity-building, and peers take on greater importance for them [36]. Nowadays, communication mediated by technology has become an important component in maintaining relationships and well-being in families, especially with teenagers [34]. Communication between parents and children influences adolescent development and can have a major impact on the development of internet addiction [37]. Adolescents who enjoy good communication with their parents can communicate more effectively and openly about their problems and are more confident [38].

This study aims to: (i) determine the prevalence of internet addiction and daytime sleepiness in the sample under study; (ii) explore the differences in internet addiction, family communication, and daytime sleepiness according to the participants’ sociodemographic variables such as gender and age; (iii) analyze the differences in family communication according to the level of internet addiction; (iv) analyze the differences in internet addiction according to the presence or absence of daytime sleepiness in the sample; (v) explore the association between internet addiction, family communication, and daytime sleepiness; and (vi) analyze the predictive role of gender, family communication, and daytime sleepiness for internet addiction.

## 2. Methods

### 2.1. Study Design

This is a quantitative, cross-sectional study, as the aim is to quantify phenomena using statistical procedures, and the data were collected at a single point in time. It is also a correlational study as it aims to explore the association between the variables analyzed.

### 2.2. Participants

A total of 340 adolescents aged between 12 and 17 (*M* = 14.55; *SD* = 1.69) years old participated in the study. The adolescents belonged to one high school in the north of Portugal. Among the study sample, 188 (55.3%) were female and 152 (44.7%) were male. The level of education of the adolescents ranged between the 7th and the 12th grade (14.4% were 7th grade, 21.2% 8th grade, 15.9% 9th grade, 16.5% 10th grade, 16.5% 11th grade, and 15.6% were 12th grade). 

### 2.3. Procedure

Initially, authorization was requested from the Ethics Committee of the University of Trás-os-Montes and Alto Douro (protocol code Doc107-CE-UTAD-2022, 24 November 2022). After receiving an opinion, a request for authorization was made to the Directorate-General for Education (DGE), via the Monitoring of School Surveys platform, so that the questionnaires could be administered in the school. The data were collected in one public school in the north of Portugal. A meeting was held with the school directors to choose which classes would participate. The school director randomly chose three classes from each year (7th to 12th grade). The choice of grades was in line with the age group defined in the investigation (12 to 17 years old). After that, class directors were contacted to acquire informed consent from the students. Later, consent forms were given to the parents. This defines the objectives of the study and guarantees all ethical principles, describing the voluntary nature, anonymity, and confidentiality of the investigation. After collecting the informed consent forms, a date was agreed to administer the questionnaires to the students. The students who granted permission were then given the questionnaires and were informed in detail about the aims of the study, the voluntary nature of their participation, and their right to withdraw from the study at any time. The students filled out the questionnaires in the classroom, which took around 20 min to complete.

### 2.4. Measures

Sociodemographic questionnaire: The questionnaire covered participants’ demographic information, such as sex, age, and grade.

Internet Addiction Test (IAT) [9] (translated by Pontes [39]): This is a 20-item questionnaire in which respondents are asked to rate the items on a five-point Likert scale (0 = never; 1 = seldom; 2 = occasionally; 3 = frequently; 4 = very often, 5 = always), with the main aim being to assess the degree of the individual’s involvement with the internet [39]. The minimum score is 20, the maximum is 100 and the higher the score, the greater the problems internet use causes. A result of 0 to 19 indicates the absence of addiction, from 20 to 39 indicates a low level of addiction and an average online user, from 40 to 69 it represents a moderate level of addiction, while a result of 70 to 100 indicates a severe level of internet addiction [9]. Internal consistency in the present sample was acceptable, with a Cronbach’s alpha score of 0.87. Confirmatory factor analysis revealed appropriate adjustment, with acceptable fit (χ^2/df^ = 2.380; *p* = 0.005; GFI = 0.977; CFI = 0.981; RMSE = 0.064).

Pediatric daytime sleepiness scale (PDSS) [40] (translated by Moreno [41]): This scale aims to assess daytime sleepiness in children and adolescents through a self-assessment, which describes daily life situations related to sleep habits [41]. This questionnaire is composed of eight multiple choice questions, ranging from 0 to 32 points. All questions were presented in a Likert-scale format (0 = never, 1 = seldom, 2 = sometimes, 3 = frequently, 4 = always); however, the question “Are you alert/attentive most of the day?” was on a reverse scale. The point distribution for the PDSS is 0–32 and the highest score of PDSS indicated more daytime sleepiness [40]. In this study, the Cronbach alpha score was 0.88 and the confirmatory factor analysis revealed appropriate adjustment, with acceptable fit (χ^2/df^ = 1.754; *p* = 0.025; GFI = 0.977; CFI = 0.967; RMSE = 0.047).

The family communication scale (FCS) [42] (translated by Rebelo [43]): This scale assesses the positive communication that exists in a family system. The 10 items are answered on a 5-point Likert scale, from “strongly disagree” to “strongly agree” and a higher total score (range 10–50) indicates a greater level of positive family communication [43]. Regarding psychometric proprieties, the Cronbach’s alpha score in this sample was 0.70. Confirmatory factor analysis revealed appropriate adjustment indices (χ^2/df^ = 1.733; *p* = 0.006; GFI = 0.967; CFI = 0.982; RMSE = 0.047).

### 2.5. Statistical Analysis

All statistical analyses were carried out using IBM SPSS (Statistical Package for the Social Sciences—version 27) and IBM SPSS AMOS (Analysis of Moment Structures—version 21) for Windows. Initially we proceeded to clean the sample by identifying possible missing values and outliers that could undermine the study and the reliability of results. The analysis of outliers was obtained by determining Zscores. On missing values, if a participant had more than 10% of their answers missing, they were removed from the sample. Therefore, 4 participants were excluded from the sample. The dimensions of the instruments were also created, as well as the inversion of negative items based on the authors of the instruments used. Subsequently, the psychometric properties of the different instruments were analyzed through a reliability analysis carried out in SPSS and a confirmatory factor analysis carried out using AMOS. This made it possible to evaluate the reliability of the instruments, as well as their adjustment values. In line with Pallant [44], the Cronbach’s alpha coefficient value must be above 0.70. According to Marôco [45], when the sample exceeds 30 subjects, the normal distribution of a sample is accepted and parametric tests can be used. We also carried out psychometric analyses using Cronbach’s alpha and factor analysis. The tests were bilateral with a statistical significance set at 0.05 and descriptive statistics were used to determine the mean and standard deviation. Therefore, in this study, differential analyses were carried out using ANOVA, with the identification of the partial eta squared value, which, according to Cohen [46], may indicate a small effect when presenting values of 0.01, a moderate effect when presenting values of 0.06, or a large effect when presenting values of 0.14. A Pearson correlation coefficient was performed, and the magnitude of the effect was identified, low correlations were considered with values between 0.10 and 0.29, medium correlations with values between 0.30, and 0.49 and high correlations with values between 0.50 and 1.0 [46]. Since the conditions for carrying out the regression analysis were met, a hierarchical multiple regression was performed, for which it was necessary to codify the variable sex as a dummy variable, assigning zero to the female and the value of one to the male. 

## 3. Results 

### 3.1. Prevalence of Internet Addiction and Daytime Sleepiness in the Sample

According to Young’s [15] guidelines, this study found three levels of internet addiction: normal users (*n* = 122; 35.9%), users with mild addiction (*n* = 163; 47.9%) and users with moderate addiction (*n* = 55; 16.2%). In this study, no users with severe internet addiction were found (Table 1).

Regarding daytime sleepiness, the Drake [40] procedures tell us that individuals who score above 20 points have excessive daytime sleepiness and in this study 63 (18.5%) adolescents had daytime sleepiness (Table 1).

### 3.2. Differential Analysis of Internet Addiction, Family Communication, and Daytime Sleepiness According to the Sex of Adolescents

To analyze the difference between internet addiction and the sex of the adolescents, we used analysis of variance (ANOVA). The results (Table 2) showed that there were no statistically significant differences [*F*(1,338) = 0.014; *p* = 0.906; *ηp*2 = 0.000] between male adolescents (*M* = 1.78; *SD* = 0.66) and female adolescents (*M* = 1.79; *SD* = 0.69).

To analyze the difference between family communication and the sex of the adolescents, we also used analysis of variance (ANOVA). The family communication scale (Table 2) also revealed no significant differences [*F*(1, 338) = 3.211; *p* = 0.074; *ηp*2 = 0.009] between male (*M* = 4.02; *SD* = 0.59) and female (*M* = 3.90; *SD* = 0.66) participants.

To study the difference between daytime sleepiness and the sex of the adolescents, we used analysis of variance (ANOVA). The results (Table 2) showed that there are significant differences [*F*(1, 338) = 7.544; *p* = 0.006; *ηp*2 = 0.022], with females (*M* = 1.95; *SD* = 0.62) presenting higher levels of daytime sleepiness compared to males (*M* = 1.76; *SD* = 0.66).

### 3.3. Differential Analysis of Internet Addiction, Family Communication, and Daytime Sleepiness by Age

The sample was divided into two age groups based on information from the United Nations (UN): typical ages of middle school (group 1: 12–14 years old) and high school students (group 2: 15–17 years old) [47]. Using these age groupings, we conducted an analysis of variance (ANOVA) to analyze the internet addiction, family communication, and daytime sleepiness according to age.

As depicted in Table 3, there were significant differences between the two age groups in internet addiction [*F*(1, 338) = 13.834; *p* = 0.000; *ηp*2 = 0.039]. Younger adolescents reported more internet addiction (*M* = 1.93; *SD* = 0.72) than older adolescents (*M* = 1.66; *SD* = 0.60). There were no significant differences between the ages in terms of family communication (*p* = 0.331) or daytime sleepiness (*p* = 0.438) (Table 3).

### 3.4. Differential Analysis of Family Communication according to Level of Internet Addiction

To analyze the difference between family communication and the level of internet addiction, we used analysis of variance (ANOVA). According to the results (Table 4), there were statistically significant differences between the study groups (absence of addiction, low level of addiction and moderate level of addiction) in family communication [*F*(2, 337) = 19.044; *p* = 0.000; *ηp*2 = 0.102]. Due to the presence of an independent variable with 3 levels, post hoc analyses were carried out using the Scheffé test. The analyzes indicate statistically significant differences in family communication between the no addiction (*p* = 0.000), as well as between users with a low level of addiction and a moderate level of addiction (*p* = 0.000). However, there were no significant differences between users with mild and moderate levels of addiction (*p* = 0.123) (Table 4).

### 3.5. Differential Analysis of Internet Addiction according to the Presence or Absence of Daytime Sleepiness

To analyze the difference between internet addiction and the presence or absence of daytime sleepiness, we used analysis of variance (ANOVA). Observation of the results revealed statistically significant differences in the internet addiction of adolescents with or without excessive daytime sleepiness [*F*(1, 338) = 57.965; *p* = 0.000; *ηp*2 = 0.146]. Analyses of the estimated marginal means showed that adolescents with daytime sleepiness were more dependent on the internet, with a higher mean (*M* = 2.33; *SD* = 0.66) than adolescents without daytime sleepiness (*M* = 1.66; *SD* = 0.62).

### 3.6. Association between Internet Addiction, Family Communication, and Daytime Sleepiness

To verify the relationship between internet addiction, family communication, and daytime sleepiness a Pearson correlation was conducted. Regarding this analysis (Table 5) the was a negative average association between internet addiction and family communication (*r* = −0.328; *p* < 0.01). It was also verified that a positive average association existed between internet addiction and daytime sleepiness (*r* = 0.488; *p* < 0.01) and a negative low association existed between family communication and daytime sleepiness (*r* = −0.240; *p* < 0.01).

### 3.7. Predictive Analysis: The Predictor Role of Adolescents’ Sex, Family Communication, and Daytime Sleepiness in Internet Addiction

Aiming to determine which independent variables best predict internet addiction, hierarchical multiple regressions analyses were conducted (Table 6). Block 1 corresponded to the dummy variable, sex of the adolescent (being 0 for females and 1 male); Block 2 corresponded to family communication; and Block 3 to daytime sleepiness.

Block 1 explained 0% of the total variance (*R*^2^ = 0.000) not presenting a significant contribution [*F*(1, 338) = 0.014; *p* = 0.906; *R*^2^*change* = −0.003]. Block 2 explained 10.8% of the total variance (*R*^2^ = 0.108), individually contributing to 10.3% of the variance for the model (*R*^2^*change* = 0.103) and representing a significant contribution [*F*(1, 337) = 20.463; *p* = 0.000]. Block 3 explained 29.2% of the total variance (*R*^2^ = 0.286), individually contributing to 28.6% of the variance for the model (*R*^2^*change* = 0.286) and representing a significant contribution [*F*(1, 336) = 46.188; *p* = 0.000].

Analyzing the contribution of each of the variables in the blocks individually, two variables show significant contributions (*p* ≤ 0.05) as predictors of internet addiction: family communication (β = −0.331) negatively predicts internet addiction and daytime sleepiness (β = 0.470) positively predicts internet addiction. Sex is not statistically significant and does not contribute to the prediction of the dependent variable.

## 4. Discussion

The aim of this study was therefore to analyze internet addiction in adolescents and check its relationships with daytime sleepiness and family communication.

Regarding the prevalence of internet addiction, and according to Young’s [15] criteria, this study did not find any users with severe addiction. However, 64.1% (*n* = 218) of the participants had a mild or moderate dependency rate. The results are like another study [48] carried out on a sample of Portuguese adolescents aged between 14 and 18 (73.1%; *n* = 415) and the results of a study [39] carried out on a sample of Portuguese adolescents and young adults (60%; *n* = 356). In contrast, other studies [49,50] have found that 16.5% and 3.4% of participants, respectively, had severe internet addiction. The results of various studies show that internet addiction is becoming widespread among teenagers, and it is important to intervene from a preventative point of view. The high prevalence of internet addiction observed may be due to the digital inclusion policy that has made free internet access available in schools. Therefore, internet addiction in Portuguese adolescents has been shown to be more than abusive consumption and has specific criteria, including a significant impact on the young person’s life [21].

Regarding the presence or absence of daytime sleepiness in adolescents, this study found that 63 participants (18.5%) were classified as having excessive daytime sleepiness. The results are like another study [51] carried out on a sample of Portuguese adolescents, where 11% of young people met the criteria for daytime sleepiness. Another study shows a higher percentage of young people with sleep-related problems, where 46.6% of the sample was classified as having daytime sleepiness [29]. On the other hand, there are several studies analyzing daytime sleepiness with different instruments [52,53], but the results show that excessive daytime sleepiness in adolescents is a growing public health problem.

The results of sex differences in internet addiction are divergent. Our results are in line with some findings [50,54] where there were no significant differences between males and females, using the IAT. However, the literature shows several inconsistencies in the effects, since other studies [16,53] using the same instruments have produced results showing a higher prevalence of addiction in females compared to males. On the other hand, several studies have found that males have higher internet addiction scores than females, using different instruments: Young’s internet addiction test [49] and Young’s 10-item internet addiction test [55]. According to Giacometti-Rocha and Mill [56], female teenagers use the internet more to access websites about beauty, while male teenagers use the internet to play games. Also, females have higher percentages for accessing content about education and work compared to males [56]. Other studies also show that females value socialization through online social networks more, while males prefer activities related to online games [57,58]. The variety of differences by gender may be due to the numerous measuring instruments and cut-off points used to demarcate dependency levels, as well as cultural differences between countries [7]. 

In view of the results, there were significant differences regarding the sex of the adolescent about daytime sleepiness, pointing to female adolescents having a higher proportion of excessive daytime sleepiness. The same was verified in the study by Chung et al. [59], where female teenagers are sleepier during the day, as they use their cellphones more than their male colleagues. Another study also found that females feel drowsier during the day (71.3%) than males, as they feel sleepier and sleep more during the day [60].

When analyzing the difference between family communication and the adolescent’s sex, no significant differences were found. The results agree with another study [61]. According to McNaughton [62], women are more encouraged to share their emotions and feelings than men, and girls end up sharing more with their parents, so they perceive greater parental availability than young men. In the present study, while males had a slightly higher level of family communication, the average between the male groups was not significant when compared to females. This suggests that young men are beginning to show greater openness to communicating with their parents, contrary to the idea that young men are less interested in developing relationships and social ties with their relatives.

Analyzing the age of adolescents there were significant differences in internet addiction, with the 12 to 14 age group presenting higher levels of addiction than the 15 to 17 age group. The results are in line with data from various studies [7,21,49]. We should consider that older pupils have more school demands than younger pupils and may limit their use of the internet during the week. No significant differences were found between daytime sleepiness and the age of the adolescents. In contrast, other research has found interaction between ages, associating excessive daytime sleepiness with older adolescents, as they tend to sleep fewer hours a night and use technology more before going to bed, thus impairing sleep [63,64]. From a neurobiological perspective, the use of different technologies, such as computers, cellphones, and the use of the internet, has a negative impact on sleep at night, increasing drowsiness during the day [65]. Excessive daytime sleepiness also occurs when the individual engages in different tasks before going to sleep [30]. In fact, the use of technology and the internet captures adolescents’ attention, changing their waking rhythm and the number of hours of sleep, modifying their psychological well-being [26]. Thus, insufficient sleep can seriously impair cognitive and learning abilities, with the highest changes being found in children and adolescents [64]. According to the literature, the desire for autonomy and the preference for extra-familial ideas are manifested above all in young people aged 15 and over, making relationships and interactions between family members difficult [66]. In this sense, the older the adolescent becomes, the less open communication they have with their parents [67], since they have already achieved independence and autonomy and, therefore, the family is no longer the center of their attention [68]. However, in this study, no significant differences were found in terms of family communication according to age.

There were significant differences between internet addiction and the presence or absence of daytime sleepiness, indicating that adolescents with higher levels of internet addiction were sleepier during the day. This may be related to the use of the internet during the night or moments before going to bed, thus impairing sleep duration [69] and increasing daytime sleepiness the following day. Scientific evidence states that the blue light emitted by electronic devices, such as cell phones, can suppress melatonin secretion and negatively interfere with adolescent sleep [70,71]. Additionally, studies argue that procrastination before bed can be a mediator between the use of technology and sleep quality [72]. Bedtime procrastination is defined as going to bed later than intended despite the absence of external reasons [73]. Furthermore, constantly checking one’s cell phone before going to bed can interfere with an individuals’ routines due to procrastination by spending time online [72]. It has been observed that daytime sleepiness plays a predictive role in internet addiction since, as addiction increases, the likelihood of developing excessive daytime sleepiness increases. The results are in line with other studies [29,59,74,75], where it was found that excessive use of the internet by adolescents was positively associated with daytime sleepiness. This is why teenagers who are sleepier during the day prefer to carry out activities that require minimal physical effort, such as staying in front of the television, on the computer or on their cellphone [74].

The family life cycle during adolescence undergoes numerous changes that affect not only the adolescent but the entire family [76]. Communication during this phase is fundamental to the family structure, as family members share experiences that allow family relationships to be organized [77]. In this sense, the increase in internet use over the last decade has led to problematic behaviors that affect social functioning, including family relationships [78]. It was possible to observe that family communication negatively predicts internet addiction. Given this situation, it seems to us that when adolescents have a high level of internet addiction, family communication tends to be worse. In other words, good communication between parents and children leads to a lower probability of young people developing internet addiction. These results are in line with other research [78,79]. Good family communication is essential if young people are to avoid developing problems related to uncontrolled internet use [80]. In this sense, family communication acts as a preventative strategy for risky behaviors [34], which leads to the conclusion that higher levels of communication between parents and their teenage children suggests that younger children are less likely to develop behaviors associated with excessive internet use [81]. In the sample under study, there was a relationship between family communication and levels of internet addiction. The results found here are in line with the results of some other studies [82,83], which found a poorer quality of family communication among adolescents with internet addiction.

These results suggest that a family with poor communication may have a predictive effect on young people developing internet addiction. This research increases understanding of the influence of parent–child family communication on adolescents’ internet addiction and helps to identify underlying mechanisms to prevent internet addiction. In this way, positive family communication should be worked on to prevent and avoid internet-dependent adolescents.

## 5. Practical Implications, Limitations, and Recommendations for Future Research

The present study has limitations that may appear to be indications and suggestions for future studies. The sample cannot be considered representative for the Portuguese population as it was only collected in one district in the north of the country. It is a cross-section study not allowing the establishment of cause–effect relationships. Another limitation concerns the use of self-report questionnaires to measure the variables under analysis, which can lead participants to manipulate or respond in accordance with social desirability. The lack of clarity about the boundary between excessive and inappropriate internet use is an issue that plagues research and continues to be one of the main challenges in defining the concept of internet addiction [84]. Finally, it is important to mention that the cut-off points presented by Young [15] for the IAT instrument are merely speculative, since no clinical studies were carried out to prove them, and it is essential to interpret the results with caution [39].

Regarding recommendations for future research, we suggest better exploring the dimension of family communication and its relationship with internet addiction and highlighting the importance of carrying out longitudinal studies to contribute to the perception of the development of internet addiction. Finally, internet addiction is strongly associated with excessive daytime sleepiness, and this translates into a reduction in physical exercise by adolescents, harming their physical and psychological health [63], so it is important to delve deeper studies on the same.

## 6. Conclusions

This study showed that internet addiction is a growing problem in adolescent health. In addition, there is an association between internet addiction and daytime sleepiness. It is important to instill healthy sleep habits and to develop preventive interventions and strategies to reduce excessive daytime sleepiness in adolescents. The growth of the internet and technological advances have had an impact on communication, changing not only adolescents’ means of communicating with peers, but also family communication patterns. It is essential to create good family communication, since this is a preventative factor for internet addiction in teenagers. In this sense, the free time of adolescents is poorly distributed, which facilitates the practice of sedentary activities, thus making young people aware of the importance of physical exercise is essential. Therefore, it is important to have a careful assessment of young people’s exposure to the internet and their sleeping habits to establish adequate strategies to prevent this problem. Thus, the practice of physical activities is recommended, as these help in the development of the adolescent, reduce the risks of future diseases, and encourage a healthier lifestyle.

## Figures and Tables

**Table 1:** Prevalence Indicators of Internet Addiction and Daytime Sleepiness.

Variables		Absolute Frequency	%
Internet addiction	Absence of addiction	122	35.9%
	Low level of addiction	163	47.9%
	Moderate level of addiction	55	16.2%
Daytime sleepiness	Without daytime sleepiness	277	81.5%
	With daytime sleepiness	63	18.5%

**Table 2:** Differential Analysis of Internet Addiction, Family Communication and Daytime Sleepiness According to the Sex of Adolescents.

	Sex	*M ± SD*	95% CI	Direction of Significant Differences
Internet addiction	1-Male	1.78 ± 0.65	[1.68, 1.89]	--
	2-Female	1.79 ± 0.69	[1.69, 1.89]	
Family communication	1-Male	4.02 ± 0.59	[3.93, 4.12]	--
	2-Female	3.90 ± 0.66	[3.80, 3.99]	
Daytime sleepiness	1-Male	1.76 ± 0.66	[1.66, 1.87]	2 > 1
	2-Female	1.95 ± 0.62	[1.87, 2.04]	

*M* = mean, *SD* = standard-deviation, 95% CI = 95% confidence interval.

**Table 3:** Differential Analysis of Internet Addiction, Family Communication, and Daytime Sleepiness According to the Age of Adolescents.

	Age	*M* ± *SD*	95% CI	Direction of Significant Differences
Internet addiction	1–12 to 14	1.93 ± 0.72	[1.81, 2.04]	1 > 2
	2–15 to 17	1.66 ± 0.60	[1.57, 1.75]	
Family communication	1–12 to 14	3.92 ± 0.64	[3.82, 4.02]	--
	2–15 to 17	3.99 ± 0.62	[3.89, 4.08]	
Daytime sleepiness	1–12 to 14	1.90 ± 0.68	[1.79, 2.00]	--
	2–15 to 17	1.84 ± 0.61	[1.75, 1.93]	

*M* = mean, *SD* = standard-deviation, 95% CI = 95% confidence interval.

**Table 4:** Differential Analysis of Family Communication According to Level of Internet Addiction.

	Level of Addiction on the Internet	*M* ± *SD*	95% CI	Direction of Significant Differences
Family communication	1-Absence of addiction	4.21 ± 0.53	[4.11; 4.30]	1 > 2 > 3
	2-Low level of addiction	3.86 ± 0.61	[3.77; 3.96]	
	3-Moderate level of addiction	3.67 ± 0.71	[3.47; 3.86]	

*M* = mean, *SD* = standard-deviation, 95% CI = 95% confidence interval.

**Table 5:** Association Between Internet Addiction, Family Communication, and Daytime Sleepiness.

	1	2	3	*M* ± *SD*
1-Internet addiction	-			1.79 ± 0.68
2-Family communication	−0.328 *	-		3.95 ± 0.63
3-Daytime sleepiness	0.488 *	−0.240 *	-	1.87 ± 0.64

*M* = mean, *SD* = standard-deviation, * *p* < 0.01.

**Table 6:** The Predictor Role of Adolescents’ Sex, Family Communication, and Daytime Sleepiness for Internet Addiction.

Internet Addiction	*R* ^2^	*R* ^2^ *Change*	B	SE	β	*t*	*p*
Block 1- Dummy Sex	0.000	−0.003	0.009	0.074	0.006	0.118	0.906
Block 2- Family Communication	0.108	0.103	−0.354	0.055	−0.331	−6.396	0.000
Block 3- Daytime Sleepiness	0.292	0.286	−0.470	0.050	0.445	9.337	0.000

B, S. error and β for a significance level of *p* < 0.05.

## Data Availability

The data are not publicly available due to privacy.
